# Temporal Trends in *Bordetella pertussis* Populations, Denmark, 1949–2010

**DOI:** 10.3201/eid1805.110812

**Published:** 2012-05

**Authors:** Randi Føns Petersen, Tine Dalby, Ditte Marie Dragsted, Frits Mooi, Lotte Lambertsen

**Affiliations:** Statens Serum Institut, Copenhagen, Denmark (R.F. Petersen, T. Dalby, L. Lambertsen);; private practitioner, Frederiksberg, Denmark (D.M. Dragsted);; National Institute for Public Health and the Environment (RIVM), Bilthoven, the Netherlands (F. Mooi)

**Keywords:** Bordetella pertussis, multilocus variable-number tandem repeat analysis, MLVA, multiple antigen sequence typing, MAST, prn, ptxA, ptxP, tcfA, vaccination, whooping cough, Denmark, bacteria

## Abstract

We used multilocus variable-number tandem repeat analysis and multiple antigen sequence typing to characterize isolates of *Bordetella pertussis* strains circulating in Denmark during periods with and without pertussis vaccination coverage. Our results show substantial shifts in the *B. pertussis* population over time and a reduction in genetic diversity. These changes might have resulted from the introduction of pertussis vaccines in Denmark and other parts of Europe. The predominant strains currently circulating in Denmark resemble those in other European countries.

## Abstract 2

Reduced genetic diversity possibly resulted from introduction of pertussis vaccines

Whooping cough is a vaccine-preventable disease caused by the bacterium *Bordetella pertussis*; however, the protection conferred by vaccination does not last throughout life. In Denmark, as in other industrialized countries with high vaccination coverage, the disease is still endemic despite ≈50 years of vaccination. In 1961, a whole-cell *B. pertussis* (wP) vaccine was introduced into the childhood vaccination schedule in Denmark. In January 1997, it was replaced by a monocomponent acellular *B. pertussis* (aP) vaccine (DiTeKiPol or DiTeKiPol/Act-Hib; Statens Serum Institut [SSI], Copenhagen, Denmark) containing hydrogen peroxide–inactivated *B. pertussis* toxoid as the sole pertussis antigen. This vaccine was originally described and patented by the National Institutes of Health (Bethesda, MD, USA) (*1**,**2*). In 2003, a booster dose for preschool-age children (5 years of age) was introduced (diTekiBooster or DiTeKiPol Booster; SSI). Coverage among infants in Denmark for the third dose of pertussis vaccine has traditionally been high (≈87%–91%) (*3*). Acellular pertussis vaccines can contain up to 5 different antigens from *B. pertussis*, and Denmark is the only country using a monocomponent vaccine for both primary and booster vaccination (*4*).

The incidences of whooping cough and related deaths in Denmark have decreased dramatically since the introduction of pertussis vaccines. During a whooping cough epidemic in the early 1950s, before vaccine was introduced, the incidence of infection was ≈11,000 per 100,000 infants (*5*). In 2010, however, the incidence of infection among infants 0–1 year old had dropped to 110 per 100,000 infants and the incidence among the whole population had dropped to 7 per 100,000 persons (*6*). In addition, the last 2 pertussis–related deaths among infants in Denmark were notified in 2010 and 2005 (*7*). In general, the population-wide incidence of whooping cough in Denmark has been low since implementation of the pertussis vaccination program. However, occasional epidemic peaks have occurred, and the latest 2 were in 2002 (incidence, 36 cases/100,000 persons) and 2004 (incidence, 24 cases/100,000 persons) (*8*).

To determine the predominant strains of *B. pertussis* circulating in Denmark, we characterized clinical isolates obtained during 3 periods. Period 1, comprised 1949–1961, the year vaccine was introduced; period 2 comprised 1962–1996, during which wP vaccine was used; and period 3 comprised the years after 1996, during which aP vaccine has been used. We characterized the isolates by using multilocus variable-number tandem repeat analysis (MLVA) and multiple antigen sequence typing (MAST) to partially sequence the genes encoding pertactin (*prn*), *B. pertussis* toxin S1 subunit (*ptx*A, also designated *ptxS1*), *B. pertussis* toxin promoter (*ptxP*), and tracheal colonization factor A (*tcfA*). Because there is no consensus about which genes should be included in MAST of *B. pertussis,* we designated the results obtained in Denmark as MASTdk.

## Materials and Methods

### Strain Collection

*B. pertussis* isolates were available from the strain collection at SSI. Strains in the collection for 1950–1974 were lyophilized; for 1974–1994, isolates were either lyophilized or stored in liquid nitrogen, and after 1995, they were mostly stored at −80°C. The isolates were cultured on *B. pertussis* charcoal agar plates containing cephalexin and 10% horse blood (SSI). Plates were incubated at 36°C for up to 5 days.

### Selection Criteria

Strains were selected to cover whooping cough patients from all over Denmark during 1 prevaccine and 2 postvaccine periods. A total of 24 isolates were selected from period 1 (1961 and earlier); 51 were selected from period 2 (1962–1996); and 185 were selected from period 3 (1997 forward). Strains were selected regardless of the patient’s sex, age, clinical complications, and vaccine history; limited or no information was known about the patients.

### DNA and Primers

Approximately 1 μL (1 inoculation loop) from a pure 4-day culture of *B. pertussis* on agar was suspended in 200 μL of sample preparation reagent (PrepMan; Applied Biosystems, Foster City, CA, USA), boiled for 10 min, and centrifuged for 10 min at maximum speed. For PCR, 2 μL of a 1:400 dilution of lysate was used. Primers used in the study are shown in Table 1.

**Table 1:** Primers used for MLVA and MASTdk typing in a study of temporal trends in *Bordetella pertussis* populations, Denmark, 1949–2009*

Primer name	Target	Sequence, 5′ → 3′	Reference
BP-VNTR1-DF	VNTR1	FAM-CCTGGCGGCGGGAGACGTGGTGGTG	(*9*)
BP-VNTR1-DR	VNTR1	AAAATTGCGGCATGTGGGCTGACTCTGA	(*9*)
BP-VNTR3-BF	VNTR3	FAM -GCCTCGGCGAAATTGCTGAAC	(*9*)
BP-VNTR3-BR	VNTR3	GCGGGCGAGGAAACGCCCGAGACC	(*9*)
BP-VNTR4-CF	VNTR4	FAM-CGTGCCCTGCGCCTGGACCTG	(*9*)
BP-VNTR4-BR	VNTR4	GCCGCTGCTCGACGCCAGGGACAA	(*9*)
BP-VNTR5-BF	VNTR5	FAM-GAAGCCGGCCCACCCGAGCTCCAGGCTCTT	(*9*)
BP-VNTR5-BR	VNTR5	TGCCGGGTTTCGGCATCTCGATGGGATACG	(*9*)
BP-VNTR6-EF	VNTR6	FAM-CCAACGGCGGTCTGCTGGGTGGTC	(*9*)
BP-VNTR6-FR	VNTR6	AGGGCGCTGGTCACGCCACCGAGGAT	(*9*)
BP-PtxP-F	Pertussis toxin promoter	AATCGTCCTGCTCAACCGCC	(*10*)
BP-PtxP-R	Pertussis toxin promoter	GGTATACGGTGGCGGGAGGA	(*10*)
BP-Ptx S1-F2	Pertussis toxin Subunit 1	CCCCCTGCCATGGTGTGATC	(*11*,*12*)
BP-Ptx S1-R2	Pertussis toxin Subunit 1	AGAGCGTCTTGCGGTCGATC	(*11*,*12*)
BP-prn-AF	Pertactin region 1	GCCAATGTCACGGTCCAA	(*11*–*13*)
BP-Prn-AR	Pertactin region 1	GCAAGGTGATCGACAGGG	(*11*–*13*)
BP-Prn-BF	Pertactin region 2†	AGCTGGGCGGTTCAAGGT	(*11*–*13*)
BP-Prn-BR/	Pertactin region 2†	CCGGATTCAGGCGCAACTC	(*11*–*13*)
BP-tcfAF	Tracheal colonization factor	TTCTTGCGCGTCGTGTCTTC	(*9*)
BP-tcfAR3	Tracheal colonization factor	GCGGTTGCGGACCTTCAT	(*9*)

*MLVA, multilocus variable-number tandem repeat analysis; MASTdk, multiple antigen sequence typing results obtained in Denmark; VNTR, variable-number tandem repeat. †Pertactin region 2 was sequenced to confirm *prn* allele type (1 or 7).

### MLVA

Six loci, originally identified by Schouls et al. (*9*), were included in the typing of isolates: variable-number tandem repeats (VNTRs) 1, 3a, 3b, 4, 5, and 6. VNTR3b is a duplication of VNTR3a and is present only in a subset of isolates.VNTR2 was excluded from the analysis because it did not exhibit substantial variation between isolates (*9*). Amplification was performed in a total volume of 20 μL; 2 μL of a 1:400 dilution of lysate was added to a mix of 1 μL of each primer (10 pmol/μL), 10 μL of HotStarTaq Master Mix Kit (QIAGEN, Hilden, Germany), and 4 μL of 5M betaine (Sigma-Aldrich Chemie, Zwijndrecht, the Netherlands) for VNTR1, 3, 4, and 5 or 6 μL of 5 M betaine for VNTR 6.

The PCR protocol we used was a modified version of protocols published by Schouls et al. (*9*) and Kurniawan et al. (*14*). PCRs were set up as monoplex reactions and amplified in similar PCR programs, except that the annealing temperature was 68°C for primers 1, 5, and 6 and 60°C for primers 3–4. Amplification was initiated by denaturation at 96°C for 15 min and followed by 40 cycles at 95°C for 20 s, 68°C or 60°C for 30 s, 72°C for 90 s, and a final extension step at 72°C for 20 min. Final PCR products of VNTR 1, 5, and 6 and VNTR 3 and 4 were diluted 1:100 and 1:200, respectively, before the fragments were separated on an ABI 3130 DNA analyzer (Applied Biosystems).

### Data Analysis

We converted the DNA analyzer–determined size of each VNTR into the number of repeat units by using a custom-made conversion table. Data were imported to BioNumerics version 6.1 software (Applied Maths, Sint-Martens-Latem, Belgium) and analyzed. Each isolate was defined by an MLVA profile containing a string of numbers representing the number of repeats at each allele in the following order: VNTR1, 3a, 3b, 4, 5, and 6. Each unique MLVA profile was assigned an MLVA type (MT) and named according to the Dutch scheme (*15*).

To verify the correct conversion from sequence sizes to number of repeats, we sequenced 1–3 representatives of each VNTR size. Sequencing results showed that there is a difference between the size determined by fragment analysis and the size obtained by sequencing in our setup of the ABI 3130 DNA analyzer. VNTR1 (15-bp unit) had a difference of 26 bp, VNTR3 (5-bp unit) 10 bp, VNTR4 (12 bp-unit) 3–4 bp, VNTR5 (6 bp-unit) 9–11 bp, and VNTR6 (9-bp unit) 9–12 bp (data not shown). In all cases, the sequencing size was larger than the DNA analyzer–determined size, and the difference for each VNTR was stable for all investigated sizes. Schouls et al. suggested that the inaccurate sizing obtained by the DNA analyzer resulted from the secondary structure of the PCR product (*9*).

### MAST (MASTdk)

Four genetic loci known to be polymorphic for *B. pertussis* were selected for the MASTdk analysis: *ptxP* (*9**–**11*), *ptxA* (*11**,**12*), and 2 genes encoding surface proteins, tracheal colonization factor A (*tcfA*) (*9**,**11*) and pertactin (*prn*) (*11**–**13*). Sequence typing was performed as described for the individual genes (Table 1).

### Bioinformatics

MLVA profiles were clustered in the BioNumerics version 6.1 software by using a categorical coefficient and visualized by using the minimum spanning tree method. Temporal tendencies of MLVA and MAST were determined by using the statistics tool in the BioNumerics program. The genetic diversity (Simpson’s index of diversity) was calculated by using Comparing Partitions, an online tool for quantitative assessment of partition congruence (http://www.comparingpartitions.info).

## Results and Discussion

### MLVA

We used MLVA to type 260 clinical *B. pertussis* isolates collected in Denmark during 1949–2010. The isolates were resolved into 40 MTs, 27 of which have been published (*15*). These new types derived from the entire study period. Two MTs, MT27 and MT29, were predominant among all isolates investigated, representing 47% and 19% of the isolates, respectively. We found 7 MTs in >4 isolates, and 31 were in 1–3 isolates only and were compiled into a single group, denoted minor types, that represented ≈20% of all types (Table 2). The allelic profiles of all MTs found in this study are summarized in the Table A1).

**Table 2:** MLVA typing results for isolates analyzed in a study of temporal trends in *Bordetella pertussis* populations, Denmark, 1949–2009*

MT	No. isolates detected (% frequency)			
	Total period, n = 260 isolates, 40 MTs	Period 1, n = 24 isolates, 8 MTs	Period 2, n = 51 isolates, 17 MTs	Period 3, n = 185 isolates, 23 MTs
Major MTs				
27	122 (47)	ND	3 (6)	119 (65)
29	49 (20)	3 (12)	27 (53)	19 (10)
36	8 (3)	ND	ND	8 (4)
253†	7 (3)	7 (29)	ND	ND
34	6 (2)	ND	2 (4)	4 (2)
141	5 (2)	4 (17)	1 (2)	ND
18	4 (1)	ND	ND	4 (2)
256†	4 (1)	4 (17)	ND	ND
16	4 (1)	ND	1 (2)	3 (2)
Minor MTs‡	51 (20)	6 (25)	17 (33)	28 (15)

*Genetic diversity was calculated as Simpson index of diversity for each period. The genetic diversity index was calculated for MLVA types by using the online tool Comparing Partitions (www.comparingpartitions.info). Simpson index of diversity (95% CI) was 0.74 (0.69–0.79) for the total period, 0.84 (0.75–0.92) for period 1, 0.71 (0.57–0.85) for period 2, and 0.57 (0.48–0.65) for period 3. MLVA, multilocus variable-number tandem repeat analysis; MT, MLVA type; ND, none detected. †MTs found in 1–3 isolates. ‡New MTs detected in this study.

**Figure 1:**
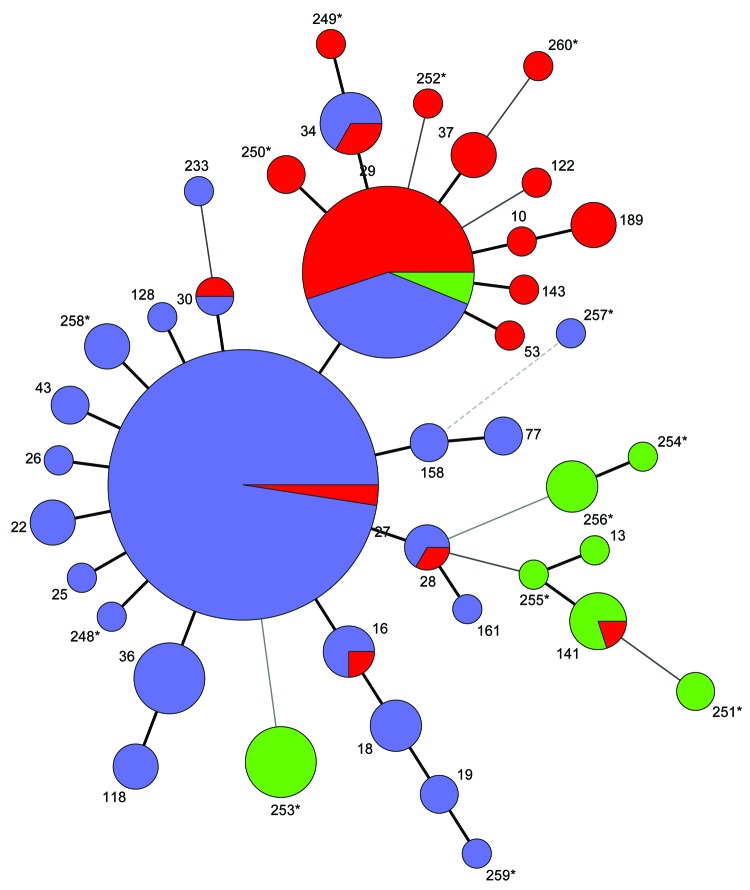
Multilocus variable-number tandem repeat analysis types (MT) of 260 *Bordetella pertussis* isolates collected in Denmark during 1949–2010. A minimum spanning tree based on the categorical clustering of MTs. Each MT is represented by a circle, and the Dutch type name (*15*) is indicated. MTs connected by heavy short lines, thinner lines, dashed lines, and spotted lines designate 1, 2, 3, and 4 loci differences, respectively. *New MTs detected in this study. Circle size indicates the number of isolates with the particular MT. Colors indicate the time period of isolation: green, period 1 (1961 and earlier); red, period 2 (1962–1996); purple, period 3 (1997 forward).

**Figure 2:**
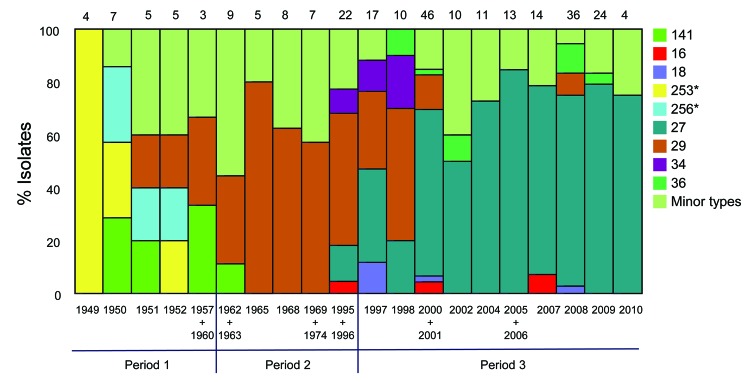
Temporal trends in the frequency of multilocus variable-number tandem repeat analysis types (MTs) of *Bordetella pertussis* isolates collected in Denmark, 1949–2010. MTs represented in 1–3 isolates are compiled into 1 group denoted minor types. Years containing 1 or 2 isolates are combined with another year in the same time period, as indicated. The number of isolates analyzed each year/years is given above each column.

The clonal relationship between different MTs was investigated and visualized by constructing a minimum spanning tree based on the categorical clustering of MLVA profiles (Figure 1). The tree showed that the majority of isolates belonged to 1 of the dominant MTs, MT27 or MT29, or were single-locus variants to 1 of these. MT27 and MT29 differed from each other by a single locus. A smaller proportion of isolates were more divergent, showing multiple locus differences between the MTs. The more divergent MTs primarily derived from period 1, and most isolates from period 2 were MT29 or single-locus variants of this type; period 3 was dominated by MT27 or single-locus variants of this type (Figure 2; Table 2). MT29 was first detected in Denmark in 1951, and during period 2, the proportion of MT29 increased to constitute 53% of isolates during that period. During period 3, MT27 gradually replaced MT29 to become the predominant type, constituting 65% of the isolates in period 3.

We determined the genetic diversity (Simpson’s index of diversity) of MLVA for periods 1, 2, and 3 to be 0.84, 0.72, and 0.57, respectively (Table 2). These numbers reflect a tendency of decreasing genetic diversity from period 1 to 3, i.e., a change from a more even distribution of isolates among the detected MTs toward single dominant types (Figure 2). Throughout the 3 study periods, we detected a proportion of minor and new MTs, which indicated the continuous appearance of new genetic *B. pertussis* types, regardless of the use or type of vaccine. Our results are in line with observations from the Netherlands (*9*), the United Kingdom (*16*), and Australia (*14*).

### MAST

We analyzed the following genes of a selection of *B. pertussis* isolates by using previously published sequencing methods: *prn* (153 isolates), *ptxA* (151 isolates), *ptxP* (148 isolates), and *tcfA* (153 isolates). These genes were selected because they are the most polymorphic genes found in *B. pertussis*, and we were particularly interested in variation in *PtxA* and the *Ptx* promoter because Denmark has used a monocomponent vaccine containing only pertussis toxoid since 1997. Before this study, 13 *prn*, 8 *ptxA* (*17*), 4 *tcfA* (*11*), and 14 *ptxP* (*10**,**18*) alleles had been identified. In our study, we detected 4 *prn* alleles (*prn1*, *prn2*, *prn3,* and *prn7*), 3 *ptxA* alleles (*ptxA1*, *ptxA2*, and *ptxA4*), 5 *ptxP* alleles (*ptxP1*, *ptxP2*, *ptxP3*, *ptxP15*, and *ptxP17*), and 2 *tcfA* alleles (*tcfA*2 and *tcfA*3).

**Figure 3:**
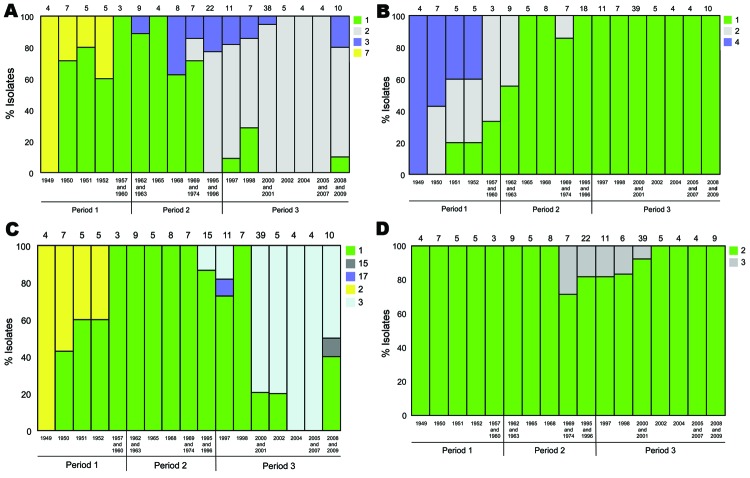
Temporal trends in the frequencies of the alleles of pertactin (*prn*) (A), the pertussis toxin subunit S1 (*ptxA*) (B), the pertussis toxin promoter (*ptxP*) (C), and the tracheal colonization factor A (*tcfA*) (D) of *Bordetella pertussis* isolates collected in Denmark, 1949–2010. The number of isolates analyzed each year/years is given above each column. Years containing 1 or 2 isolates are combined with another year in the same time period, as indicated

We analyzed the frequencies of the individual alleles and their temporal trends (Table 3; Figure 3). In the case of *prn* (Figure 3, panel A), there was a replacement of predominant alleles from period 1 to periods 2 and 3. The *prn1* allele dominated in period 1 and 2 but was rarely observed in period 3. The *prn2* allele appeared in period 2 and dominated in period 3 (86%). The *prn3* allele was present at a low level in period 2 and 3, and the *prn7* allele was present in period 1 but was not found later.

**Table 3:** Results of partial sequencing of isolates analyzed in a study of temporal trends in *Bordetella pertussis* populations, Denmark, 1949–2009*

Partially sequenced gene, alleles detected†	No. isolates (% frequency)			
	Total period	Period 1	Period 2	Period 3
*prn*	153 (100)	24 (100)	50 (100)	79 (100)
* prn1*	41 (27)	15 (63)	22 (44)	4 (5)
* prn2*	86 (56)	ND	18 (36)	68 (86)
* prn3*	17 (11)	ND	10 (20)	7 (9)
* prn7*	9 (6)	9 (38)	ND	ND
*ptxA*	151 (100)	23 (100)	47 (100)	80 (100)
* ptxA1*	125 (83)	3 (13)	42 (89)	80 (100)
* ptxA2*	14 (9)	8 (35)	5 (11)	ND
* ptxA4*	12 (8)	12 (52)	ND	ND
*ptxP*	148 (100)	24 (100)	44 (100)	80 (100)
* ptxP1*	82 (55)	12 (50)	42 (96)	28 (35)
* ptxP2*	12 (8)	12 (50)	ND	ND
* ptxP3*	52 (35)	ND	2 (4)	50 (63)
* ptxP15*	1 (1)	ND	ND	1 (1)
* ptxP17*	1 (1)	ND	ND	1 (1)
*tcfA*	153 (100)	24 (100)	51 (100)	78 (100)
* tcfA2*	141 (92)	24 (100)	45 (88)	72 (92)
* tcfA3*	12 (8)	0	6 (12)	6 (8)

*ND, none detected. †Genes encoding pertactin (*prn*), *B. pertussis* toxin S1 subunit (*ptx*A), *B. pertussis* toxin promoter (*ptxP*), and tracheal colonization factor A (*tcfA*).

We observed a similar replacement of dominant alleles for *ptxA*. Three alleles were detected in period 1: *ptxA1* (13%), *pxtA2* (35%), and *ptxA4* (52%). In period 2, *ptxA4* was not detected, *ptxA2* decreased to 11%, and *ptxA1* increased to 89%. In period 3, the *ptxA1* allele was the only allele detected. For *tcfA*, the *tcfA2* allele dominated throughout all 3 periods (Figure 3, panel D). The *tcfA3* allele was detected in few isolates during 1969–2001. In the case of *ptxP*, the *ptxP1* allele gradually replaced *ptxP2* during period 1, and *ptxP2* was not found in isolates collected after 1952. The *ptxP1* allele dominated in period 2 (96%); however, in 1995 the *ptxP3* allele was detected and increased to constitute 63% of all isolates in period 3. Sporadic isolates of *ptxP15* and *ptxP17* were detected in period 3.

Similar observations of the temporal changes of *prn* and *ptxA* alleles have been reported from other European countries, such as United Kingdom (*16*), Finland (*19**,**20*), France (*20*), and the Netherlands (*11**,**12*). The current population of *B. pertussis* in Europe is now dominated by the *prn2* allele and almost exclusively the *ptxA1* allele (*16**,**20**,**21*).

The recent emergence and current dominance of the *ptxP3* allele has also been reported for other European countries (*9**,**10**,**22**,**23*). In the Netherlands, *ptxP3* strains produced more *B. pertussis* toxin than did *ptxP1* strains*;* in addition, the emergence of *ptxP3* was associated with increased whooping cough notifications and a shift in disease prevalence toward older age groups (*10*). In Denmark, however, the emergence of *ptxP3* has not been associated with increased whooping cough notification, although a shift in age distribution has been observed (*6*). The emergence of *ptxP3* strains is unrelated to the introduction of acellular vaccines because in Denmark and the Netherlands, these strains circulated when wP vaccines were used. In the Netherlands, *ptxP3* strains reached frequencies of >90% when the wP vaccine was used for the primary series (*10*). The changes in the *B. pertussis* population in Denmark appear to be independent of epidemics because isolates from the 2002 and 2004 epidemics follow the ongoing trends in the period.

### MASTdk

**Figure 4:**
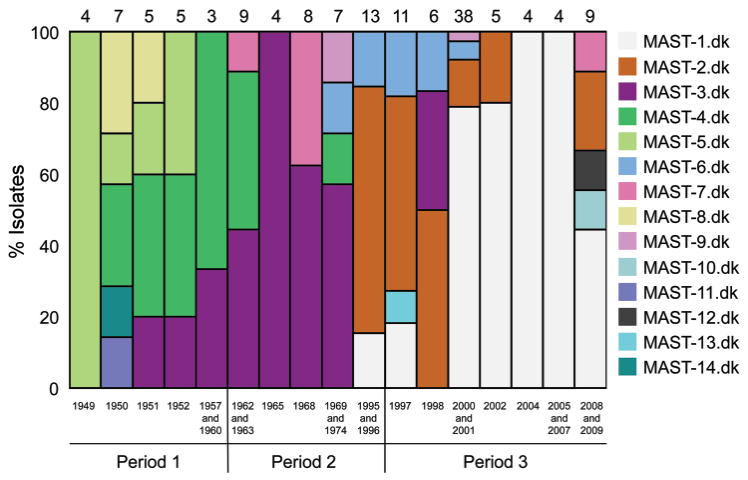
Temporal trends in the frequency of multilocus sequence type types (MASTdk) of *Bordetella pertussis* isolates collected in Denmark, 1949–2010. Years with 1 or 2 isolates are combined with another year in the same time period, as indicated. The number of isolates analyzed each year/years is given above each column.

We identified 14 combinations of the sequence types of *prn*, *ptxA*, *ptxP*, and *tcfA* (MASTdk); some MASTdk types appeared to be dominant and were detected in multiple isolates, whereas others were seen in few or a single isolate (Table 4). Similar to our MLVA findings, we found that the genetic diversity determined by using Simpson’s index of diversity based on the MASTdk results decreased from 0.78 in period 1 to 0.77 in period 3 to 0.56 in period 3 (Figure 4; Table 4).

**Table 4:** MASTs detected in isolates analyzed in a study of temporal trends in *Bordetella pertussis* populations, Denmark, 1949–2009*

MASTdk	Allele types in MASTdk					No. isolates (% frequency)†			
	*prn*	*ptxA*	*ptxP*	*tcfA*		Total period, n = 142	Period 1, n = 24	Period 2, n = 41	Period 3, n = 77
MAST1.dk	2	1	3	2		50 (35)	ND	2 (5)	48 (63)
MAST2.dk	2	1	1	2		26 (18)	ND	9 (22)	17 (22)
MAST3.dk	1	1	1	2		22 (15)	3 (13)	17 (42)	2 (3)
MAST4.dk	1	2	1	2		13 (9)	8 (33)	5 (12)	ND
MAST5.dk	7	4	2	2		8 (6)	8 (33)	ND	ND
MAST6.dk	3	1	1	3		8 (6)	ND	3 (7)	5 (7)
MAST7.dk	3	1	1	2		5 (3)	ND	4 (10)	1 (1)
MAST8.dk	1	4	2	2		3 (2)	3 (13)	ND	ND
MAST9.dk	2	1	1	3		2 (1)	ND	1 (2)	1 (1)
MAST10.dk	2	1	15	2		1 (1)	ND	ND	1 (1)
MAST11.dk	7	2	2	2		1 (1)	1 (4)	ND	ND
MAST12.dk	3	1	3	2		1 (1)	ND	ND	1 (1)
MAST13.dk	1	1	17	2		1 (1)	ND	ND	1 (1)
MAST14.dk	1	4	1	2		1 (1)	1 (4)	ND	ND

*Sequence types are given in the order *prn-ptxA-ptxP-tcfA*. MASTs, multilocus sequence types; MASTdk, MAST types detected in Denmark during 1949–2010. ND, none detected. †Genetic diversity index calculated on multilocus variable-number tandem repeat analysis types. Calculations were done by using the online tool Comparing Partitions (www.comparingpartitions.info). Simpson’s index of diversity (95% CI) was 0.80 (0.76–0.84) for the total period, 0.77 (0.68–0.86) for period 1, 0.76 (0.67–0.85) for period 2, and 0.56 (0.45–0.67) for period 3.

The trends for *B. pertussis* MTs and sequence types (or MAST) are similar in Denmark and the Netherlands (*9**,**10**,**23*). Different vaccines have been used in these countries, which may suggest that the most important factor driving these changes is not the type of vaccine used, but the removal, by vaccination, of immunologically naive infants as a major source of *B. pertussis* transmission. This might, as suggested (*10*), have selected for strains that are more efficiently transmitted by adolescents and adults, in whom immunity has waned.

## Conclusions

By using MLVA and sequence typing of *B. pertussis* isolates collected in Denmark during 1949–2010, we showed that the population of this pathogen has changed over time. In general, the *B. pertussis* population in Denmark has changed from having a higher genetic diversity, as measured by MLVA and sequence typing, toward the dominance of single types. The predominant MLVA- and MASTdk-identified strains currently circulating in Denmark resemble the types observed in other European countries.

The genetic diversity of the *B. pertussis* isolates in Denmark was highest during period 1 (1949–1961), i.e., before introduction of pertussis vaccine. Period 2, when wP vaccine was used, was dominated by few or single types, as is the case in period 3, when aP vaccine has been used. The observed genetic changes of *B. pertussis* could therefore be related to the introduction of vaccines. However, there is no evidence that the wP or aP vaccines used in Denmark have selected for other dominant MT or sequence types than those observed in other European countries. Also, since the aP vaccine used in Denmark contains only *B. pertussis* toxoid, changes in the *prn* alleles in period 3 must have occurred independently of that vaccine. Travel within Europe has increased substantially since the prevaccine era; thus, *B. pertussis* imports from neighboring countries might explain the shift in the *B. pertussis* populations in Denmark. This explanation is supported by the appearance of similar *B. pertussis* types around Europe and by the occurrence of such types in Denmark before the introduction of change in pertussis vaccines in Denmark. The true explanation for the changes in genetic diversity among *B. pertussis* isolates in Denmark is probably a combination of those 2 theories.

## Appendix

**Table A1:** MLVA type and allelic profiles in a study of temporal trends in *Bordetella pertussis* populations, Denmark, 1949–2009*

MLVA type	No. isolates	Allelic profile
Major†		
27	122	8-7-0-7-6-7
29	49	8-7-0-7-6-9
36	8	8-7-8-7-6-7
253§	7	10-4-0-7-14-7
34	6	8-7-0-8-6-9
141	5	7-7-0-8-6-8
18	4	8-6-7-7-6-7
256§	4	10-5-0-7-13-8
16	4	8-6-0-7-6-7
Minor‡		
118	3	8-7-8-8-6-7
189	3	7-7-0-7-5-9
22	3	8-7-0-6-6-7
258§	3	8-7-0-7-8-7
28	3	8-7-0-7-6-8
37	3	8-7-8-7-6-9
158	2	8-7-0-7-7-7
19	2	8-6-7-7-6-9
250§	2	8-7-0-9-6-9
251§	2	7-8-0-8-6-7
30	2	8-7-0-7-6-10
43	2	9-7-0-7-6-7
77	2	8-6-0-7-7-7
10	1	7-7-0-7-6-9
122	1	8-8-0-7-7-9
128	1	5-7-0-7-6-7
13	1	7-7-0-9-5-8
143	1	8-7-0-5-6-9
161	1	9-7-0-7-6-8
233	1	9-7-0-5-6-10
248§	1	8-7-0-4-6-7
249§	1	8-7-0-8-6-1
25	1	8-7-0-7-5-7
252§	1	6-6-0-7-6-9
254§	1	10-5-0-7-15-8
255§	1	7-7-0-9-6-8
257§	1	2-1-0-4-7-5
259§	1	8-6-7-8-6-9
26	1	8-7-0-7-6-6
260§	1	4-7-8-7-7-9
53	1	8-7-0-6-6-9

*MLVA, multilocus variable-number tandem repeat analysis. †MLVA type found in >4 isolates. ‡MLVA type found in <3 isolates. §New MLVA type detected in this study.
